# Mathematical Model Reveals the Role of Memory CD8 T Cell Populations in Recall Responses to Influenza

**DOI:** 10.3389/fimmu.2016.00165

**Published:** 2016-05-09

**Authors:** Veronika I. Zarnitsyna, Andreas Handel, Sean R. McMaster, Sarah L. Hayward, Jacob E. Kohlmeier, Rustom Antia

**Affiliations:** ^1^Department of Microbiology and Immunology, Emory University School of Medicine, Atlanta, GA, USA; ^2^Department of Epidemiology and Biostatistics, College of Public Health, University of Georgia, Athens, GA, USA; ^3^Department of Biology, Emory University, Atlanta, GA, USA

**Keywords:** recall response, influenza, T cell, resident memory, central memory

## Abstract

The current influenza vaccine provides narrow protection against the strains included in the vaccine, and needs to be reformulated every few years in response to the constantly evolving new strains. Novel approaches are directed toward developing vaccines that provide broader protection by targeting B and T cell epitopes that are conserved between different strains of the virus. In this paper, we focus on developing mathematical models to explore the CD8 T cell responses to influenza, how they can be boosted, and the conditions under which they contribute to protection. Our models suggest that the interplay between spatial heterogeneity (with the virus infecting the respiratory tract and the immune response being generated in the secondary lymphoid organs) and T cell differentiation (with proliferation occurring in the lymphoid organs giving rise to a subpopulation of resident T cells in the respiratory tract) is the key to understand the dynamics of protection afforded by the CD8 T cell response to influenza. Our results suggest that the time lag for the generation of resident T cells in the respiratory tract and their rate of decay following infection are the key factors that limit the efficacy of CD8 T cell responses. The models predict that an increase in the level of central memory T cells leads to a gradual decrease in the viral load, and, in contrast, there is a sharper protection threshold for the relationship between the size of the population of resident T cells and protection. The models also suggest that repeated natural influenza infections cause the number of central memory CD8 T cells and the peak number of resident memory CD8 T cells to reach their plateaus, and while the former is maintained, the latter decays with time since the most recent infection.

## 1 Introduction

Influenza A is a vaccine preventable disease that still causes substantial morbidity and mortality (1). Currently approved vaccines aim at boosting antibody responses to major influenza surface proteins hemagglutinin (HA) and neuraminidase (NA). These antigens constantly mutate resulting in antigenic drift and requiring annual update to the virus strains in the vaccine. Furthermore, the current vaccination approach leaves the population almost completely unprotected following much larger antigenic changes in the influenza virus called antigenic shifts (for example, from H1N1 to H2N2) that are associated with pandemics (2, 3).

The major focus of research and vaccination has been on antibody responses to the HA and NA proteins of influenza, which exhibit considerable evolution. Experimental studies suggest that CD8 T cell and antibody responses provide two independent responses that seem to be redundant to some extent (4–7). In contrast to epitopes that are targeted by antibody responses, the CD8 T cell epitopes on the virus are largely conserved between influenza strains within a given subtype and even between different subtypes (8–11). Thus, CD8 T cell immunity generated from an infection with one influenza strain might provide some protection following a challenge with a new strain. This has been shown in animal model systems following subsequent infections with two heterosubtypic strains of influenza as well as by adoptive transfer experiments (12–15). Challenge of an animal that has recovered from an infection with one influenza strain with a heterosubtypic (i.e., shifted) strain allows us to assess the contribution of T cells to protection because of the lack of cross-reactive antibodies between the influenza viruses of different subtypes. Several experimental studies have shown that infection of mice with one strain of influenza can lessen the disease caused by infection with heterosubtypic strains (12–15). In particular, infection of mice and non-human primates with seasonal influenza can protect against challenge with a heterosubtypic pandemic strain (14, 16, 17). A second line of evidence comes from the adoptive transfer experiments. It has been shown that the adoptive transfer of large numbers of memory CD8 T cells, generated following H3N2 influenza infection, into naive congenic mice can provide some protection following infection with a pandemic H1N1 strain (12).

Studies in the animal model systems described above have clearly shown that T cell-mediated protection is in principle possible to achieve, and this is supported by human studies. Retrospective studies have shown that prior infection of humans with the seasonal H1N1 strain provided some protection to the pandemic H2N2 strain in 1957 (18). Furthermore, the level of preexisting influenza-specific cytotoxic T cells was associated with a lack of viral shedding 4–5 days after inoculation of human volunteers with influenza virus, indicating faster virus clearance, although there was no clear association with influenza-related symptoms (19).

In this paper, we use mathematical models to explore how repeated influenza infections affect the generation of CD8 T cell immunity, how this immunity wanes with time, and how protection against recall influenza infections depends on the magnitude of this immunity. The models consider the key features of the interplay between the virus and the CD8 T cell response. We include spatial heterogeneity as the location of the infection in the respiratory tract is different from the secondary lymphoid organs where the CD8 T cell response is generated. Furthermore, modeling the dynamics of generation of responses requires incorporation of the different populations of CD8 T cells and their migration between the secondary lymphoid organs and the respiratory tract where the infection is localized.

We specifically use the models to ask the following questions. First, what determines the dynamics of the virus and the different subpopulations of CD8 T cells in the lymph nodes and respiratory tract following primary infection? Second, how does the number of influenza-specific CD8 T cells in the lymph nodes and respiratory tract decay following the clearance of the infection? Third, how is protection related to the number of influenza-specific CD8 T cells in the secondary lymphoid organs and respiratory tract? Fourth, what affects the dynamics of virus and CD8 T cells during recall responses and, in particular, how do repeated infections boost CD8 T cell immunity and why do not they generate long-term protection from all new influenza infections?

## 2 Materials and Methods

### 2.1 Mathematical Model Formulation

**Figure 1:**
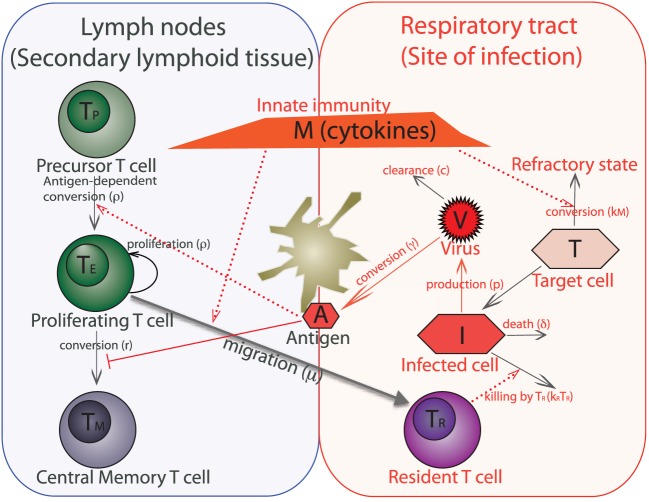
**Schematic of a within-host model of influenza infection**. The model variables are target (uninfected) epithelial cells (*T*), infected epithelial cells (*I*), virus titer (*V*), innate immunity (cytokines) (*M*), antigen presented by dendritic cells (*A*), and four populations of CD8 T cells such as virus-specific precursor cells (*T_P_*), proliferating cells (*T_E_*), central memory cells (*T_M_*), and cells which are resident at the respiratory tract (*T_R_*). We do not separately model dendritic cells, instead we consider them together with antigen as one variable. An immune response to the influenza virus mainly occurs in the two compartments – lining of the respiratory tract, which is the actual site of infection and secondary lymphoid tissue (lymph nodes) where expansion of virus-specific T cells occurs.

The models consider the key features of the interplay between the virus and the CD8 T cell response. The basic model is shown schematically in Figure 1. The immune response to the influenza virus mainly occurs in the two compartments – the respiratory tract as the actual site of infection and secondary lymphoid organs, such as lymph nodes, where the expansion of influenza-specific CD8 T cells occurs. The model also includes the target cells (epithelial cells) in which the virus can replicate, the virus, and the key subpopulations of CD8 T cells in the lymph nodes and resident cells in the lungs.

We model the dynamics of the virus and innate immunity along the lines previously described (20–22). Briefly, at the site of infection, free virus (*V*) can infect susceptible target epithelial cells (*T*), generating infected cells (*I*), which produce new virus particles [see equations (1)–(3)]. The term *βTV* represents the rate of infection of susceptible target cells by free virus. Infected cells activate innate immunity, which differs from adaptive immunity in being a saturable response (having maximum scaled to unity). The rate of activation of innate immunity depends on the number of infected cells and is half-maximal when *I* = *ϕ_M_* [equation (4)]. Innate immunity (*via* type I interferons) causes uninfected cells to become refractory to infection (23) at rate *k_M_*. Based on earlier models, we assume that during the timescale of an acute infection, the production of new target cells can be neglected, and that refractory state do not revert back to the susceptible state. The initial number of target cells in an adult’s upper respiratory tract was previously estimated as 4 × 10^8^ (20). We assume no death of target cells on the short scale of influenza infection, but infected cells have reduced lifespan in comparison to the target cells, and their lifespan is described by parameter *δ*^−1^ (see Table 1 for other model parameters).

**Table 1:** **Model parameters unless otherwise specified in the figure legend**.

Model parameter	Symbol	Units	Value
Virus infectivity	*β*	TCID_50_ ml^−1^ day^−1^	3 × 10^−05^
Virus production per cell	*p*	TCID_50_ day^−1^	0.04
Rate of virus clearance	*c*	Day^−1^	3
Infected-cell lifespan	*δ*^−1^	Day	1
Rate of killing of infected cell by *T_R_*	*k_R_*	Cells^−1^ day^−1^	0.007
Rate of conversion to refractory state	*k_M_*	Cells^−1^ day^−1^	4
Max. activation rate for innate	*σ_M_*	Day^−1^	1
Number of infected cells for half-max activation of *M*	*ϕ_M_*	Cells	1
Decay rate for innate immunity	*d_M_*	Day^−1^	0.2
Rate of virus conversion to antigen	*γ*	Day^−1^	0.3
Rate of antigen decay	*d_A_*	Day^−1^	1.7
T cell proliferation rate	*ρ*	Day^−1^	2.15
Antigen for half-maximum	*ϕ*	TCID_50_ ml^−1^	50
proliferation
Rate of migration to site of infection	*μ*	Cells^−1^ day^−1^	1.2
Rate of conversion *T_E_* to *T_M_*	*r*	Day^−1^	0.07
Rate of apoptosis for *T_E_*	*α*	Day^−1^	0.4
Death rate of *T_R_*	*d_R_*	Day^−1^	0.1

The T cell proliferation occurs in response to antigen in the secondary lymphoid organs. We assume that the rate at which antigen is brought into lymph node by dendritic cells is proportional to the amount of virus at the site of infection [see Figure 1 and equation (5)]. The number of influenza-specific CD8 T cells in the lymph nodes at the onset of infection, *T_P_*, includes both naive and memory T cells, and they are recruited into the population of proliferating cells, *T_E_*, at rate proportional to the amount of antigen $\frac{A}{\phi +A}$. The *T_E_* population grows by clonal expansion in an antigen-dependent manner (i.e., at per capita rate $\rho \frac{A}{\phi +A}$). Some of the proliferating T cells migrate to the respiratory tract by sensing the cytokines generated through activation of innate immunity *M* and become resident T cells *T_R_*. The *T_R_* cells decay at rate *d_R_*. As the antigen is cleared (i.e., at rate proportional to ($1-\frac{A}{\phi +A}$)), the *T_E_* population contracts by apoptosis at per capita rate *α* and differentiates into long-lived memory cells *T_M_* at per capita rate *r*. During recall responses, the preexisting influenza-specific central memory T cells are incorporated by changing the number of precursor cells, and we neglect the differences in the recruitment of naive and memory cells into *T_E_*.

With this assumptions, the dynamics of response to influenza infection can be described by the following differential equations:

(1) $$\left(\text{target}\;\text{cells}\right)\;\;\frac{\mathrm{dT}}{\mathrm{dt}}=-\beta \mathrm{TV}-k_{M}\mathrm{MT}$$

(2) $$\left(\text{infected}\;\text{cells}\right)\;\;\frac{\mathrm{dI}}{\mathrm{dt}}=\beta \mathrm{TV}-k_{R}T_{R}I-\delta I$$

(3) $$\left(\text{viral}\;\text{titer}\right)\;\;\frac{\mathrm{dV}}{\mathrm{dt}}=\mathrm{pI}-\mathrm{cV}$$

(4) $$\left(\text{innate}\right)\;\;\frac{\mathrm{dM}}{\mathrm{dt}}=\frac{\sigma_{M}I}{\left(\phi_{M}+I\right)}\left(1-M\right)-d_{M}M$$

(5) $$\left(\text{antigen}\right)\;\;\frac{\mathrm{dA}}{\mathrm{dt}}=\gamma V-d_{A}A$$

(6) $$\left(\text{presursor}\right)\;\;\frac{dT_{P}}{\mathrm{dt}}=-\rho T_{P}\left(\frac{A}{\phi +A}\right)$$

(7) $$\begin{array}{ll} \left(\text{expanding}\right)\;\;\frac{dT_{E}}{\mathrm{dt}} & =\rho \left(T_{P}+T_{E}\right)\left(\frac{A}{\phi +A}\right)-\left(\alpha +r\right)T_{E}\left(1-\frac{A}{\phi +A}\right)-\mu T_{E}M \end{array}$$

(8) $$\left(\text{memory}\right)\;\;\frac{dT_{M}}{\mathrm{dt}}=rT_{E}\left(1-\frac{A}{\phi +A}\right)$$

(9) $$\left(\text{resident}\right)\;\;\frac{dT_{R}}{\mathrm{dt}}=\mu T_{E}M-d_{R}T_{R}$$

We focus on immune response to acute infection, which is different from the response during persistent infections that involve very different T cell differentiation mechanisms as has been shown for CMV infection (24, 25). Our simple model for the differentiation and migration of CD8 T cells following influenza infections captures the key features of the response and is robust to many details of the pathways of T cell differentiation and to variation in parameters in biologically reasonable ranges (see Figures S1 and S2 in Supplementary Material). There are several controversies in the area of T cell differentiation and lineage relationship of CD8 T cell subsets (26–29). Our model phenomenologically captures the observation that following the response, a fraction $\frac{r}{\alpha +r}$ of the population at the peak survive as long-lived memory cells, and, consequently, is robust to the details of the underlying differentiation pathways.

We would like to note that as we focus on the role of CD8 T cells, we consider secondary infection only with heterosubtypic strain of influenza. In this case antibodies, developed during the primary response do not cross-react with the new virus strain.

## 3 Results

### 3.1 Dynamics of Primary Immune Response

**Figure 2:**
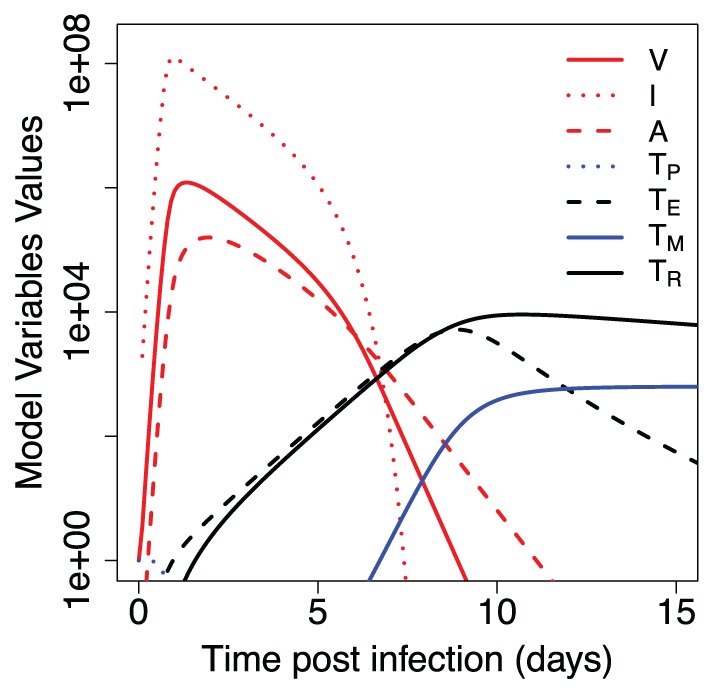
**Dynamics of primary immune response to influenza infection**. Model parameters and initial values for model variables are shown in Tables 1 and 2.

Figure 2 shows the results of our model for the dynamics of primary immune response to the influenza. The virus undergoes an expansion phase following a contraction phase. As in previous modeling studies (20–22), the peak of the virus is largely controlled by available target cells and innate immunity. T cells proliferate and a fraction of them migrate to the respiratory tract, where they kill the virus-infected cells and help to eliminate the infection. There is a delay in generation of primary CD8 T cell response due to separate spatial locations of virus entrance and place where corresponding processed antigen stimulates T cell proliferation. Proliferating CD8 T cells migrate back to the site of infection. They reach a sufficient number to affect the virus dynamics around day 6–7 and augment the innate immune system-mediated virus control. After virus clearance, expanded T cells undergo a contraction phase and develop a central memory T cell pool. Proliferation and subsequent contraction of virus-specific precursor cells in response to primary infection results in about 2–3 orders of magnitude increase in central memory T cells (*T_M_*). Resident T cells at the site of infection initially follow the dynamics of expanding cells *T_E_*, but have a slower rate of contraction after the virus clearance. The qualitative features described above are relatively robust to changes in the parameters within the biologically reasonable regime chosen (see Figure S1 in Supplementary Material).

**Table 2:** **Initial values for model variables unless otherwise specified in the figure legend**.

Model variable	Symbol	Units	Initial value
Target (uninfected) epithelial cells	*T*	Cells	4 × 10^8^
Infected epithelial cells	*I*	Cells	0
Viral titer	*V*	TCID_50_ ml^−1^	1
Innate immunity	*M*	Normalized	10^−6^
Antigen	*A*	TCID_50_ ml^−1^	0
Precursor T cells	*T_P_*	Cells	1
Proliferating T cells	*T_E_*	Cells	0
Central memory T cells	*T_M_*	Cells	0
Resident T cells	*T_R_*	Cells	0

*These values represent the “naive” state, before first influenza infection encounter. Initial value for precursor T cells is normalized by the number of virus-specific CD8 T cells and thus equal to 1*.

**Figure 3:**
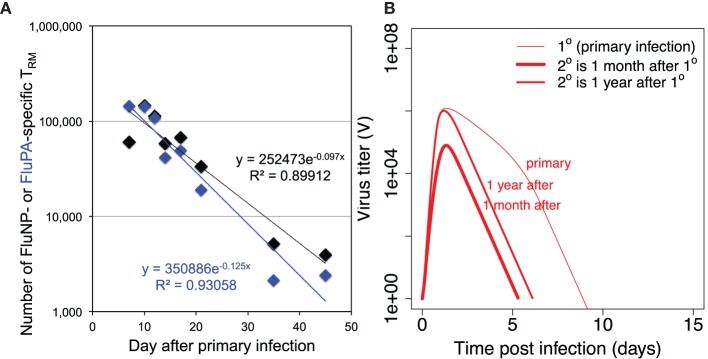
**(A)** shows the dynamics of loss of resident CD8 T cells after primary infection and estimation of the value of parameter *d_R_* (the rate of decay of resident T cells) from the data on mice intranasally infected with primary influenza A virus strain A/HKx31 (H3N2) at 30,000 50% egg infectious dose (EID_50_). Numbers of lung resident CD8 T cells specific for influenza epitopes FluNP and FluPA were measured at indicated time points. Each data point is the average from 5 to 20 mice. All experiments were completed in accordance with the Institutional Animal Care and Use Committee guidelines of Emory University. **(B)** shows the dynamics of virus during primary versus recall infections when recall infection happens 30 days or 1 year after the first infection. For secondary infections the initial values for *T_P_* and *T_R_* were taken from the corresponding values of variables *T_P_* + *T_M_* and *T_R_*, respectively, in simulation of primary infection with other parameters and initial values as in Tables 1 and 2.

Two T cell populations are left after primary response. Central memory T cells *T_M_* are known to have low level of decay (30), so we assume no decay rate for them in the model. The decay rate of resident memory T cells *T_R_* is described by parameter *d_R_* in the model. We estimated its value from the data on the primary influenza A infection in mice (Figure 3A). The decay rate for resident CD8 T cells at the respiratory tract of humans is unknown, and in our model, we assume its value to be similar to the one estimated in mice.

### 3.2 Dynamics of Secondary Immune Response

Figure 3B shows the dynamics of the virus when secondary infection occurs 1 month or 1 year after the primary infection. Several observations can be made. First, during secondary infection, the achieved maximum of virus titer is always lower than in primary infection. Second, the extended time between the infections leads to less reduction in the level of virus replication in comparison to primary infection. Third, the duration of secondary infection is shorter by a couple days in both cases. As our model does not consider waning of CD8 central memory T cells, the observed difference in the achieved virus peak values and duration of infections in Figure 3B is due to a loss of resident memory T cells between 1 month and 1 year after the primary infection.

**Figure 4:**
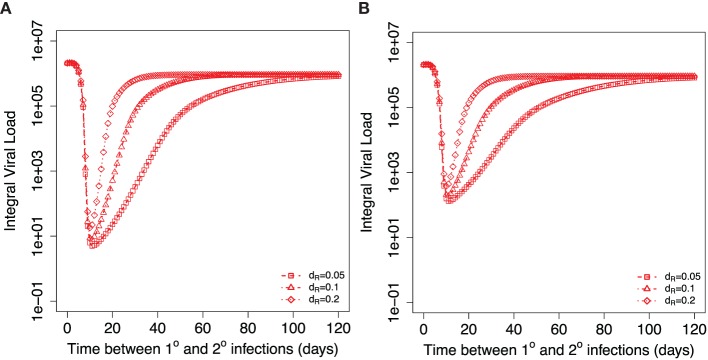
**Dependance of integral viral load achieved during secondary influenza infection on the time between primary and recall infections**. Different symbols show three different indicated values for the model parameter *d_R_* describing the decay of resident T cells at the site of infection. **(A)** shows the case when resident memory T cells are able to kill the virus infected cells immediately after the onset of secondary infection. **(B)** considers the case when resident T cells require activation to be able to kill virus-infected cells. Corresponding parameters are *k_act_* = 0.2 TCID_50_^−1^ ml day^−1^, *k_RM_* = 0.2, *ϕ_R_* = 1.

Next, we dissect, in more detail, the role of central memory T cells and resident T cells in the dynamics observed in Figure 3B. Figure 4A shows how the integral viral load changes when secondary infection occurs at different time intervals after the primary infection. For the initial conditions at the beginning of secondary infection, we take the corresponding values of *T_M_* and *T_R_* from the same time point of simulations of primary infection. The brief period of integral viral load value below 10 could be considered as strain-transcending immunity. In this case, the amount of resident T cells that exhibit cytotoxic activity at the site of infection is sufficient to prevent the infection. After the clearance of primary infection, an increase in the time between primary and secondary infections leads to a decrease in the level of resident memory T cells at the beginning of secondary infection and to a corresponding monotonic increase in the integral viral load until it reaches a plateau level. This plateau is set by the level of central memory T cells established after primary infection. Three curves on Figure 4A correspond to the different values of parameter *d_R_*. Equal integral viral load values on three curves during their raising phases correspond to equal levels of resident memory T cells at the onset of the corresponding secondary infections.

### 3.3 Effect of Additional Requirement of Activation of Resident T Cells

We also consider the case where resident T cells require time for activation before they can respond to a secondary virus challenge. We modified the equations for infected cells (*I*) and *T_R_* accordingly and added an equation for activated resident T cells (*T_RA_*):

(10) $$\left(\text{infected}\;\text{cells}\right)\;\;\;\frac{\mathrm{dI}}{\mathrm{dt}}=\beta \mathrm{TV}-k_{R}T_{\mathrm{RA}}I-\delta I$$

(11) $$\begin{array}{ll} \left(\text{resident}\right)\;\;\;\frac{dT_{R}}{\mathrm{dt}} & =-d_{R}T_{R}-k_{\mathrm{act}}T_{R}V+k_{\mathrm{RM}}T_{\mathrm{RA}}\frac{V}{\left(\phi_{R}+V\right)} \end{array}$$

(12) $$\begin{array}{ll} \left(\text{activated}\;\text{resident}\right)\;\;\frac{dT_{\mathrm{RA}}}{\mathrm{dt}} & =\mu T_{E}M+k_{\mathrm{act}}T_{R}V-d_{R}T_{\mathrm{RA}}-k_{\mathrm{RM}}T_{\mathrm{RA}}\frac{V}{\left(\phi_{R}+V\right)} \end{array}$$

Figure 4B shows the model simulations similar to Figure 4A but adjusted for the case of required activation for resident T cells. The delay in resident T cell killing activity modifies the integral viral load if secondary infections occur during second and third weeks after primary infection leading to an increase in the integral viral load. The effect of activation is less for the later times of the introduction of second virus. The gradual loss of protection with time is still determined by the rate of the decline of the resident memory T cell population. We would like to note that the integral viral load curves are mostly affected when secondary infection occurs 2–3 weeks after primary infection, and at these time points, we expect that resident T cells are still not converted into memory state. This allows us to conclude that there might be a brief period of sterilizing immunity immediately after the first influenza infection due to the resident T cells. We define sterilizing immunity as a condition with very limited or no virus replication as seen in Figures 4A and 6B.

### 3.4 Modulation of Recall Response by Prior Immunity

Assuming that each infection might boost the memory pool of CD8 T cells, a given individual at different times might have different levels of prior immunity in both central memory and resident memory compartments. The exact rules of how preexisting T cells immunity modulate an immune response to influenza challenge are unknown.

**Figure 5:**
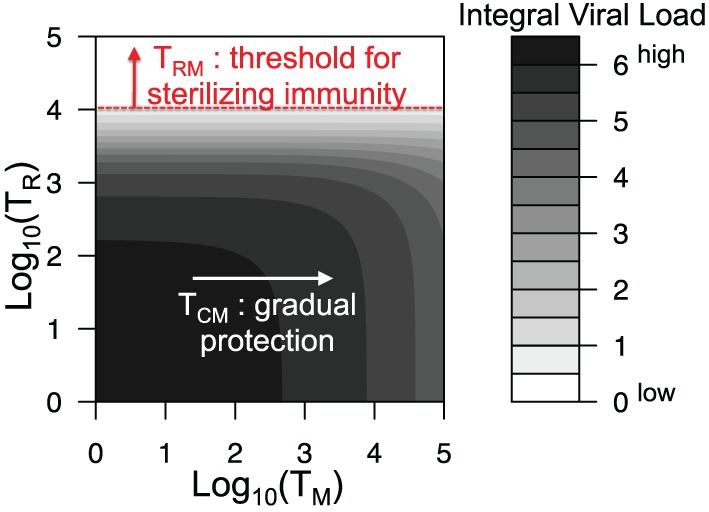
**Integral viral load dependence on the number of central memory and resident memory T cells at the onset of recall infection**. All parameters are as in Figure 2 and Table 1.

Figure 5 shows how the integral viral load depends on the level of central and resident memory CD8 T cells at the onset of recall infection in our proposed model [equation (1)–(9)]. Relatively, high level of resident T cells at the site of infection is necessary to prevent the infection. Value of parameter *k_R_* correlates with the threshold value of *T_R_* for sterilizing immunity (see Figure S2 in Supplementary Material). Central memory T cells could not prevent the infection but their increase leads to gradual protection as it reduces the integral viral load.

### 3.5 Effect of Repeated Influenza Infections on Boosting of T Cell Responses

**Figure 6:**
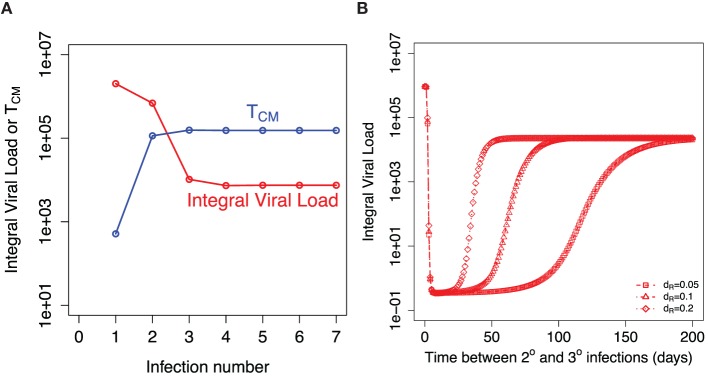
**Recall responses to sequential influenza infections**. **(A)** shows how integral viral load and number of central memory T cells (*T_M_*) depends on the number of influenza infections. The initial amount of *T_M_* for each infection is taken from the established equilibrium level values after preceding infection. **(B)** shows the dependance of integral viral load in tertiary (3°) influenza infection on the time between 2° and 3° infections. The model prediction is shown for three indicated values of the rate of loss of resident memory T cells (*d_R_*).

The key question is will repeated influenza infections keep boosting the central memory T cells and the peak of resident T cells achieved during infection or will these responses saturate? Figure 6A shows the model prediction for the integral viral load and the established level of central memory T cells after indicated number of sequential influenza infections. We assume that the next infection occurs at least 1 year after the previous one and use established level of central memory T cells from the previous infection as the initial condition for the new infection. We assume that the amount of resident T cells is significantly reduced before the new influenza season. After a few infections, both *T_M_* and integral viral load established their plateau levels. The balance between the use of existing central memory T cells and repopulation with newly formed central memory T cells is achieved.

Our model predicts that the higher level of proliferating cells during the secondary response leads to the higher level of the resident T cells. This equates to a longer period of sterilizing immunity after secondary infection. Figure 6B shows how the corresponding Figure 4A is modified when we consider the tertiary infection and plot how integral viral load depends on the time period between the second and the third influenza infections. This result is robust in a wide range of model parameters and is a consequence of higher level of central memory T cell at the beginning of the secondary infection in comparison to the primary infection.

## 4 Discussion

In this paper, we used the mathematical models to explore the role of different memory CD8 T cell populations in protection against recall influenza infections. To capture the main features of immune response to influenza a spatial heterogeneity and migration between two locations were included in the models. In the model an infection occurs in the respiratory tract, corresponding processed antigen is delivered to the secondary lymphoid organs, where it stimulates an expansion of influenza-specific CD8 T cells with subsequent migration of expanded cells to the site of infection to kill the virus. We considered specifically the role of two CD8 T cell populations in protection from recall infections: central memory T cells that predominantly reside in the secondary lymphoid organs and resident T cells in the respiratory tract. We showed that these two distinct populations can give very different protection mechanisms.

During the primary infection, the main factor determining the dynamics of CD8 T cell response is spatial heterogeneity. Antigen needs to be delivered to the secondary lymphoid organs, and expanded cells migrate back to the site of infection, which determines the timing of the response. The key factors shaping the dynamics of the recall response are the numbers of central memory and resident CD8 T cells. The model predicts that large number of resident T cells in the respiratory tract at the onset of recall infection may contribute to short-term strain-transcending immunity (Figure 5). On the contrary, the central memory T cells in the absence of resident T cells will not prevent the infection. However, an increase in their level gradually reduces the integral viral load during recall infection resulting in a quicker recovery. These model predictions are consistent with the data showing that preexisting influenza-specific CD8 T cells reduce virus titers in the lungs and trachea and protect from lethal challenge of both homologous and heterologous type A influenza viruses (12–15, 17).

Our model predicts that the key parameter to predict the longevity of T cell mediated strain-transcending immunity is the rate of loss of resident T cells in the respiratory tract. Previously, it has been shown that tissue-resident memory T cells in mice are relatively stable for 300–700 days (31–33). Interestingly, unlike resident T cells in other tissues (for example, skin resident T cells), the resident T cells in the mice respiratory tract show relatively fast decline. You can see a decrease of about two orders of magnitude in their numbers during the first month after primary influenza infection (Figure 3). In the model, we assume one population of resident memory T cells with single-exponential decay based on the available mice data.

The model predicts a longer period of strain-transcending immunity after secondary and further recall infections in comparison to primary infection. It will depend on the two factors: the level of expansion of T cells during secondary (recall) infection that determines the maximum of resident T cells achieved during infection and the further decay rate of these resident T cells. Currently, there are no mice data for the rate of decline of resident T cells in the respiratory tract after secondary infection. We assume that the rate of decline of resident T cells after secondary and further recall infections is similar to the one measured after primary infection. In this case, the model predicts that the strain-transcending immunity provided by resident memory T cells will last longer after secondary infection, but the duration of this immunity will hit a plateau level after few infections with no further increase. This reflects the initial increase with further plateau level of central memory T cells resulted from sequential infections (Figure 6).

To provide the true sterilizing immunity, the resident memory T cells should be able to immediately kill the virus-infected cells in respiratory tract tissues. This model assumption is supported by the studies showing that resident memory cells in the lungs are typically of the effector memory phenotype. They have upregulated CD69 and CD25 surface markers and lack of IL-7R expression, and might respond quickly to secondary virus challenge (34–36). We showed that relaxing this assumption and allowing some time for activation of the resident memory T cells still leads to a significant reduction in the integral viral load shortly after infection.

The model predicts that the amount of central memory T cells stabilizes after few infections. This established level in the absence of resident memory T cells still leads to a relatively large integral viral load during a recall infection. This is consistent with previous estimations that for the most of their lives adults have an influenza infection every 5 years on average (37). The extent of waning of T cell immunity is an open question. In the model, we assume that central memory T cells are maintained without decay. It has been shown that in the mice, large numbers of virus-specific memory CD8 T cells circulate through the lymphoid tissues of the previously infected animals for at least 2 years (30). The estimation of the longevity of influenza-specific T cells in humans is complicated by sampling T cells only from the blood (and not the lymph nodes) and at the current state is controversial (38, 39).

When we explored the role of resident T cells in protection (Figures 4 and 6B), we reset the level of innate immunity and target cells to their inactivated states at the beginning of a recall infection. We expect higher level of protection if the innate immunity did not revert to an inactive state or part of the cells in the respiratory tract is still in refractory or incomplete recovery states. The more detailed model accessing the role of waning of innate immunity together with the loss of resident T cells needs to be further developed.

We did not include antibody responses in our model and do not consider the role of preexisting antibody in protection during recall responses. Most of the antibody response during primary response to influenza is directed against the head region on HA. For immune response to the secondary heterosubtypic infection, we do not expect significant level of the cross-reactive antibodies as their main targeted virus proteins, specifically epitopes on the head region of HA, are substantially changed. It has been shown that antibodies to the epitopes on the stem region of HA can be broadly cross-reactive and able to recognize other subtypes (40–46). Thus, although we might neglect the contribution from these broadly cross-reactive antibodies to the stem during second infection, the repeated infections might sufficiently increase their level to facilitate the virus clearance, changing the extent of expansion of central memory T cells.

In summary, our model suggests that the resident memory CD8 T cell might contribute to the short-term strain-transcending immunity previously proposed by epidemiological studies in humans (47). In the case of repeated infections, the model predicts that the duration of strain-transcending immunity due to the resident CD8 T cells might increase in comparison to the case of primary infection and be on the scale of few months. Alternatively, preexisting antibodies at the beginning of recall infection may explain the short-term strain-transcending immunity if the rate of waning of some subsets of B-cell specific immunity is on the same scale. Both considered populations of CD8 T cells might be especially beneficial in the case of pandemic influenza in which there are no preexisting antibodies. The protective effect of central memory T cells might have a major contribution when the level of resident memory T cells is low such as at the beginning of an influenza season.

Current vaccines are not designed to induce influenza-specific cytotoxic CD8 T cells, but instead lead to boosting of the antibody responses to HA antigens that undergo drift and shift. Understanding the mechanisms underlying T cell-mediated immunity to influenza might help designing the heterosubtypic vaccines that are able to protect in the event of an unexpected pandemic challenge.

## Author Contributions

All co-authors discussed the problem, approach, and results. VZ, AH, and RA formulated the model. VZ ran simulations. SM, SH, and JK conducted the experimental studies for Figure 3A. VZ and RA wrote the paper, and all authors approved the final version.

## Conflict of Interest Statement

The authors declare that the research was conducted in the absence of any commercial or financial relationships that could be construed as a potential conflict of interest.
