# Supplementary material for: Mathematical Model Reveals the Role of Memory CD8 T Cell Populations in Recall Responses to Influenza
Source: Front Immunol. 2016 May 9;7:165. doi: 10.3389/fimmu.2016.00165 (PMC4861172; doi:10.3389/fimmu.2016.00165)
Supplement: Supplementary file 1 [file Presentation_1.pdf]

Supplementary Material: Mathematical model reveals the role of memory CD8 T cell populations in recall responses to influenza.

Veronika I. Zarnitsyna [1,*], Andreas Handel [2], Sean R. McMaster [1], Sarah L. Hayward [1], Jacob E. Kohlmeier [1] and Rustom Antia [3,*]
*Correspondence:
Veronika I. Zarnitsyna
Department of Microbiology and Immunology, Emory University School of Medicine,
1510 Clifton road, Atlanta, GA 30322, USA
vizarni@emory.edy

Rustom Antia
Department of Biology, Emory University, 1510 Clifton road, Atlanta, GA 30322,
USA rantia@emory.edu

1 SUPPLEMENTARY DATA

Model parameter ranges and outcomes

The three key parameters distinguishing our model from well-studied models of immune response to influenza are the rate of killing of infected cell by resident T cells (k_R), the rate of migration of proliferating cells to the site of infection (μ) and the death rate of resident T cells (d_R). The rest of equations were extensively studied previously. To illustrate the robustness of the main model results we used Latin Hypercube Sampling (LHS) (McKay et al., 1979), with each set of parameters shown in Supplemental Table 1. We defined the ranges for the three parameters characterizing the population of resident T cells as described below. For other model parameters we used ranges defined by 10% variation from their values reported in Table 1.

Parameters μ together with parameter ρ define the net rate of expansion of proliferating cells when the concentration of antigen is high (see equation 7 in the main text when $A \gg \phi$). Experimental data suggest expansion factor of 10^4 around day 10 after infection. Choice of the biologically reasonable range of expansion between 10^3 and 10^5 gives us the range $[1 \div 1.46]$ for parameter μ. The death rate of resident T cells was experimentally measured in mice. Decline of the resident T cells specific to FluNP and FluPA epitopes with time is reported in Figure 3 of the main text. Based on these measurements we can define the range for the parameter d_R as mean$\pm 3\times$stdev, which gives us the range $[0.05 \div 0.17]$. There is no data available for the rate of killing of infected cells by resident T cells T_R which is described by parameter k_R in the model. In the primary response, T cells arrive to the site of infection few days after the beginning of infection and the value of this parameter influences how quickly the infection will be cleared. We varied this

parameter to clear the primary infection at day 7.5 (for max value) and at day 10 (for min value). Clearance of infection was considered as equivalent to a reduction of the virus titer below the level equal to 1. That defined the range for k_R as $[0.001 \div 0.1]$. The dynamics of primary response and effect of prior immunity on the integral viral load for different combination of parameters from these biologically reasonable ranges are shown in Supplementary Figures 1 and 2, respectively.

McKay, M. D., Beckman, R. J. J, C. W., 1979 A comparison of three methods for selecting values of input variables in the analysis of output from a computer code. Technometrics 21, 239-245.

2 SUPPLEMENTARY TABLES AND FIGURES

Supplementary Table 1. Model parameters sets for the simulations in Supplementary Figures 1, 2.

Parameter	Set1	Set2	Set3	Set4	Set5	Set6	Set7	Set8	Set9
$\beta(\times 10^{-5})$	3	3.23	2.70	3.30	2.78	2.85	2.93	3.08	3.15
p	0.042	0.038	0.039	0.044	0.036	0.04	0.037	0.041	0.043
c	3.075	2.85	2.775	3.225	3.15	3	2.7	2.925	3.3
δ	0.925	1.1	1	0.95	1.075	1.025	0.975	1.05	0.9
k_R	0.1	0.003	0.018	0.01	0.056	0.032	0.002	0.001	0.006
k_M	3.7	4.1	4	4.4	4.2	3.9	3.6	4.3	3.8
σ_M	0.925	1.025	1.05	1.1	0.9	0.95	1.075	0.975	1
ϕ_M	1	0.95	1.075	1.1	0.9	1.025	0.975	1.05	0.925
d_M	0.205	0.215	0.185	0.19	0.2	0.195	0.22	0.21	0.18
γ	0.308	0.278	0.285	0.3	0.33	0.323	0.315	0.293	0.27
d_A	1.79	1.87	1.7	1.53	1.74	1.57	1.83	1.66	1.62
ρ	2.04	1.94	2.10	2.31	2.20	2.26	2.15	2.37	1.99
ϕ	51.25	50	47.5	55	46.25	53.75	52.5	45	48.75
μ	1.06	1.40	1.17	1.23	1.12	1.29	1	1.35	1.46
r	0.07	0.068	0.075	0.072	0.077	0.067	0.065	0.063	0.074
α	0.42	0.39	0.37	0.4	0.38	0.36	0.41	0.44	0.43
d_R	0.11	0.17	0.155	0.14	0.095	0.125	0.08	0.05	0.065

2.1 Figures

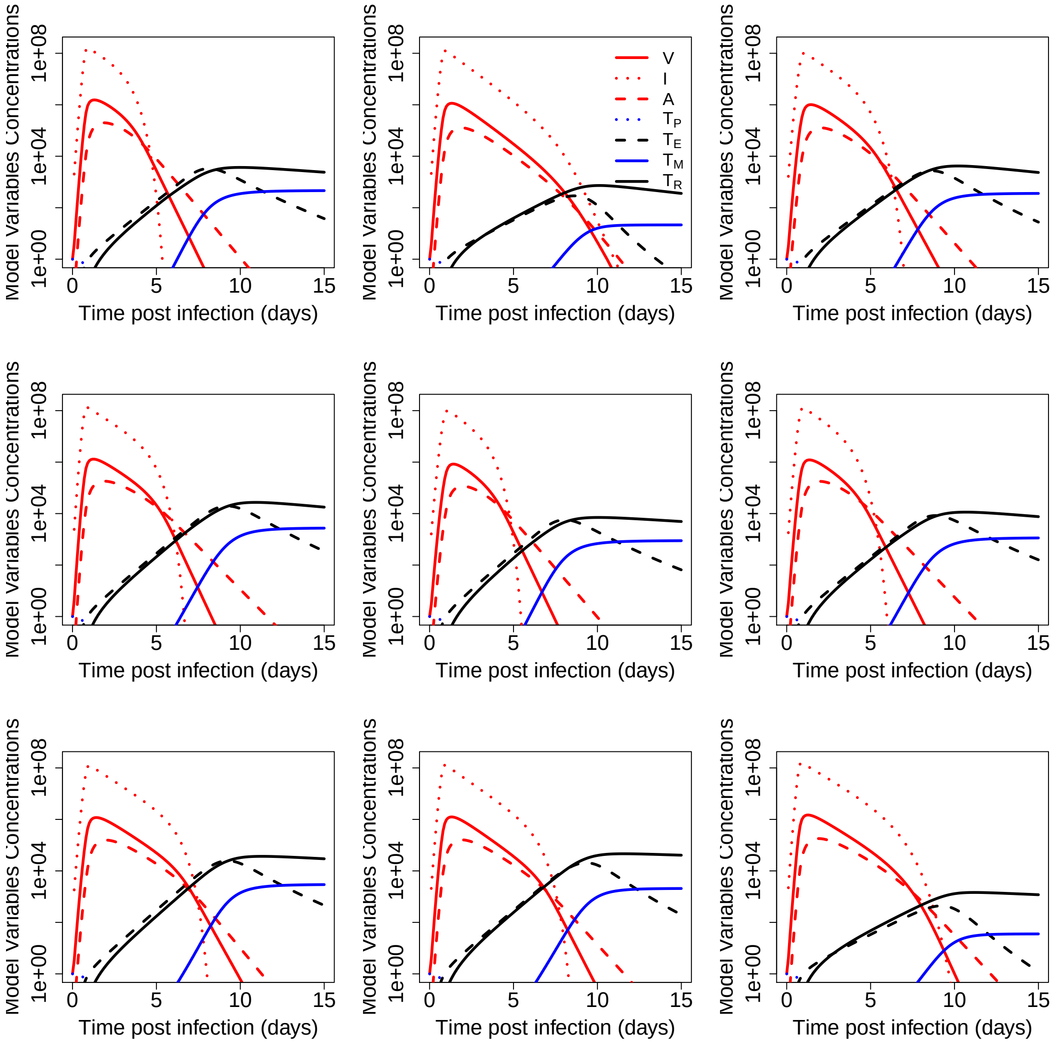

Figure S1. Dynamics of immune response to primary influenza infection. Corresponding sets of parameters are shown in Supplementary Table 1. Variables for all sets of parameters are color-coded as indicated on the panel for the parameter set 2.

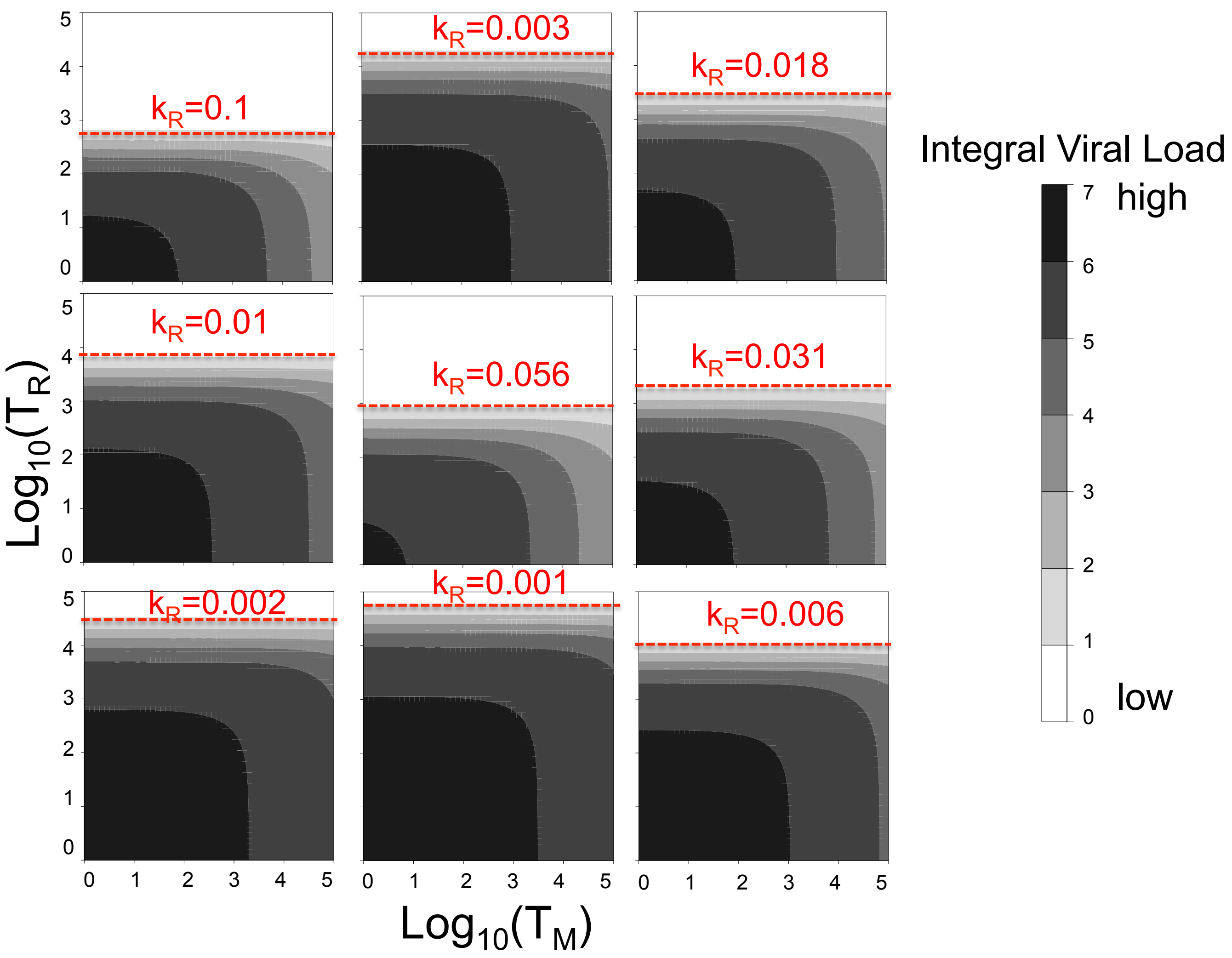

Figure S2. Dependence of Integral Viral Load on the number of central memory T_M and resident T_R cells at the onset of recall infection. Corresponding sets of parameters are shown in Supplementary Table 1. Red dashed lines show the threshold level of T_R for sterilizing immunity. Value of parameter k_R (shown above each dashed line) correlates with the threshold value of T_R for sterilizing immunity.
